# Neuron-derived exosomes-transmitted miR-124-3p protect traumatically injured spinal cord by suppressing the activation of neurotoxic microglia and astrocytes

**DOI:** 10.1186/s12951-020-00665-8

**Published:** 2020-07-25

**Authors:** Dongdong Jiang, Fangyi Gong, Xuhui Ge, Chengtang Lv, Chenyu Huang, Shuang Feng, Zheng Zhou, Yuluo Rong, Jiaxing Wang, Chengyue Ji, Jian Chen, Wene Zhao, Jin Fan, Wei Liu, Weihua Cai

**Affiliations:** 1 Department of Orthopaedics, The First Affiliated Hospital of Nanjing Medical University, Nanjing, 210029 Jiangsu China; 2 Department of Orthopaedics, Yancheng Third People’s Hospital, Yancheng, 224000 Jiangsu China; 3 Department of Orthopaedics, Nanjing First Hospital, Nanjing Medical University, Nanjing, 210006 Jiangsu China; 4 Department of Encephalopathy, The Third Affiliated Hospital of Nanjing University of Chinese Medicine, Nanjing, 210001 Jiangsu China; 5 Department of Analytical & Testing Center, Nanjing Medical University, Nanjing, 211666 Jiangsu China

**Keywords:** Spinal cord injury, Exosomes, Microglia, Astrocytes, miR-124-3p/MYH9 axis

## Abstract

**Background:**

Spinal cord injury (SCI) is a catastrophic injury that can cause irreversible motor dysfunction with high disability. Exosomes participate in the transport of miRNAs and play an essential role in intercellular communication via transfer of genetic material. However, the miRNAs in exosomes which derived from neurons, and the underlying mechanisms by which they contribute to SCI remain unknown.

**Methods:**

A contusive in vivo SCI model and a series of in vitro experiments were carried out to explore the therapeutic effects of exosomes. Then, a miRNA microarray analysis and rescue experiments were performed to confirm the role of neuron-derived exosomal miRNA in SCI. Western blot, luciferase activity assay, and RNA-ChIP were used to investigate the underlying mechanisms.

**Results:**

The results indicated that neuron-derived exosomes promoted functional behavioral recovery by suppressing the activation of M1 microglia and A1 astrocytes in vivo and in vitro. A miRNA array showed miR-124-3p to be the most enriched in neuron-derived exosomes. MYH9 was identified as the target downstream gene of miR-124-3p. A series of experiments were used to confirm the miR-124-3p/MYH9 axis. Finally, it was found that PI3K/AKT/NF-κB signaling cascades may be involved in the modulation of microglia by exosomal miR-124-3p.

**Conclusion:**

A combination of miRNAs and neuron-derived exosomes may be a promising, minimally invasive approach for the treatment of SCI.

## Background

Spinal cord injury (SCI) is a traumatic event of life-changing which leads to permanent sensory-motor disabilities, and it is estimated that the mortality rate of hospitalized acute SCI ranges from 4.4 to 16.7% globally [1]. The main causes of these injuries across most geographical locations were falls and road traffic accidents [2]. In SCI, primary damage results in death of local neurons and glia at the lesion site within minutes to hours. This is followed by secondary damage via neuroinflammatory response [3]. Inflammatory response which protects against pathogen is essential in SCI development. However, excessive inflammation may cause serious damage [4].

Functional neural competence and integrity require interactive exchanges among sensory and motor neurons, interneurons, and glial cells [5]. Microglia are the resident macrophages of the central nervous system (CNS). After SCI, microglia play a crucial role in the activation and regulation of neuroinflammation [6]. M1 microglia is considered pro-inflammatory/injury-inducing [7]. Following the number of M1 microglia increases, the ability of phagocytosis appears to significantly decrease, and there is increased secretion of inflammatory cytokines (IL-1β, IL-6, and TNF-α), chemokines, nitric oxide (NO), and reactive oxygen species (ROS) that initiate the breakdown of blood–brain barrier (BBB) [8–10]. In the case of increased BBB permeability, the secretion of chemokines by M1 microglia allow recruitment and infiltration of hematogenous leukocytes which perpetuate the inflammatory response, increasing neuronal death induced by excitotoxicity [11].

Astrocytes are abundant cells in the CNS which provide trophic support for neurons, promote synapse formation, and prune synapses by phagocytosis, in addition to fulfilling a series of other homeostatic maintenance functions [12, 13]. A1 astrocytes are induced by neuroinflammatory microglia and lose most normal astrocyte functions. A1 astrocytes obtain neurotoxic function, rapidly killing neurons and mature differentiated oligodendrocytes [14]. A1 astrocytes are highly present in human neurodegenerative diseases including Alzheimer’s, Huntington’s, Parkinson’s, and Multiple Sclerosis [14, 15]. The state induced by M1 microglia and A1 astrocytes can lead to abnormal neurotransmitter synthesis and release, synaptic destruction, and loss of other homeostatic functions [16]. Thus, efforts should focus on suppressing the activation of M1 microglia and A1 astrocytes as well as suppress detrimental excessive neuroinflammation for the treatment of SCI.

Exosomes are nano-sized vesicles released by fusion of an organelle and are important components of the paracrine secretion of cells [17]. They are formed from multivesicular bodies with a diameter of 50–150 nm [18]. Exosomes are thought to mediate a novel mechanism of cell-to-cell communication [19, 20]. Recent studies have attributed some of the tasks needed for these exchanges to extracellular vesicles, which were beneficial for SCI [21–23]. Exosomes are most prominently involved in shuttling reciprocal signals between myelinating glia and neurons, thus promoting neuronal survival, the immune response mediated by microglia, and synapse assembly and plasticity [24].

Exosomes participate in the transport of biochemicals, such as proteins, cytokines, mRNAs, and MicroRNAs (miRNAs) [25]. As a result, they play an essential role in intercellular communication through the transfer of genetic material [26, 27]. Recent studies have focused on exosomal contents, including proteins and RNAs, and attempted to determine their underlying mechanisms in the treatment of various diseases [25, 28–31]. It has been shown that exosomal miRNAs can exert their regulatory effects on target cells, thus representing a new mode of intracellular communication [18]. However, the miRNAs in exosomes derived from neurons (Exos) and the underlying mechanisms by which contribute to the functional recovery following SCI remain unknown.

We attempted to confirm a treatment role of exosomes by enhancing exosomal bioactivity through regulation of miRNAs. Using an miRNA microarray, miR-124-3p was found to be the most enriched miRNA in neuron-derived exosomes that suppressed the activation of M1 microglia and A1 astrocytes in vivo and in vitro by suppressing the activity of myosin heavy chain 9 (MYH9), thereby regulating the PI3K/AKT/NF-κB signaling cascade, and eventually promoted functional recovery following SCI in mice. This finding indicated an underlying mechanism for the application for neuron-derived exosomes and provided a promising therapeutic target for SCI.

## Methods

### Reagents and antibodies

The microglial activator lipopolysaccharide (LPS) was purchased from Sigma-Aldrich (St. Louis, MO, USA, L2630). The antibodies used for western blotting in this study included anti-GAPDH (Abcam, Rabbit, ab181602), anti-CD9 (Abcam, Rabbit, ab195422), anti-CD63 (Abcam, Rabbit, ab217345), anti-CD81 (Abcam, Rabbit, ab109201), anti-calnexin (Abcam, Rabbit, ab22595), anti-INOS (Abcam, Mouse, ab49999), anti-C3 (Abcam, Rabbit, ab97462), anti-MYH9 (Abcam, Mouse, ab55456), anti-p-PI3K (CST, Rabbit, 17366), anti-PI3K (CST, Rabbit, 4292), anti-p-AKT (CST, Rabbit, 4060), anti-AKT (CST, Mouse, 2920), anti-p-P65 (CST, Rabbit, 3033), and anti-P65 (CST, Rabbit, 8242). The antibodies used for immunofluorescence were anti-NEUN (Abcam, Rabbit, ab177487), anti-MAP2 (Abcam, Mouse, ab11267), anti-INOS (Abcam, Mouse, ab49999), anti-C3 (Abcam, Rabbit, ab97462), anti-IBA1 (Abcam, Rabbit, ab178846), anti-GFAP (Abcam, Mouse, ab10062), and anti-NF200 (Abcam, Rabbit, ab204893). and secondary antibodies were goat anti-mouse Alexa Fluor 488 (Abcam, Goat, ab150113), goat anti-rabbit Alexa Fluor 594 (Abcam, Goat, ab150088), goat anti-mouse Alexa Fluor 594 (Abcam, Goat, ab150120), goat anti-rabbit Alexa Fluor 488 (Abcam, Goat, ab150077). ELISA kits were TNF-α (R&D, MTA00B), IL-1α (R&D, MLA00), IL-1β (R&D, MLB00C), IL-6 (R&D, M6000B), and C1q (Hycult Biotech, HK211).

### Preparation of primary microglial cultures and MCM

Primary microglia were obtained as previously reported [32]. Primary microglia were removed from day 1–3 mouse brains and carefully cut into 0.5–1 mm^3^ pieces. The cut pieces were then added to the 0.25% trypsin–EDTA solution (Thermo Fisher Scientific, MA, USA) and incubated for 10 min with gentle shaking. Following termination of the trypsinization reaction, the digested tissues were centrifuged at 300×*g* for 5 min and tissue pellets were resuspended in DMEM/F12 (Gibco Laboratory, Grand Island, NY). Following filtration with a 100-μm nylon mesh, the final single-cell suspension was cultured in T75 flasks pre-coated with poly-l-lysine (Sigma-Aldrich) to obtain the primary mixed glial cell cultures. Microglia reach maturity after 14 days of culture in vitro. The mature microglia were separated by shaking at 200 rpm for 2 h at room temperature. The microglial supernatants were collected and cultured in 6- or 24-well culture plates pre-coated with poly-l-lysine at 37 °C and in 5% CO_2_-humidified atmosphere. The medium was changed every 3 days. The primary microglia were stimulated with LPS (1 μg/mL) for 24 h to induce a pro-inflammatory phenotype. Exosomes (200 μg/mL) were then added and co-cultured with the primary microglia. The conditioned medium from LPS-treated was then collected as the microglia-conditioned medium (MCM).

### Primary astrocyte cultures and treatment

Primary mixed glial cell cultures were prepared as described above. After 3 days of culture in vitro, confluent cultures were shaken at 200 rpm for 2 h on a shaker to remove microglia. The mixed glial cell cultures were then treated with astrocyte culture medium and shaken again at 240 rpm for 6 h to remove oligodendrocyte precursor cells (OPCs). The remaining astrocytes were used for further experiments. These astrocytes were then treated with MCM for 24 h to produce activated astrocytes.

### Primary neuronal cultures

The neuron culture medium was composed of neurobasal medium (Thermo Fisher Scientific), 2% B27 (Gibco Laboratory, Grand Island, NY) neurobasal supplement, 2-mM glutamine (Gibco), 1% of 100× GlutaMAX, and 1% penicillin–streptomycin. The brain dissociation procedure was similar to the microglia isolation procedure described above. The conditioned medium from these neurons was then collected as the neuron-conditioned medium (NCM) or treated with GW4869 (Sigma-Aldrich), a neutral sphingomyelinase inhibitor known to block exosome secretion [33].

### Exosome isolation and identification

Exosomes were prepared from neuronal primary culture NCM. The medium was collected and centrifuged at 300×*g* for 10 min and 2000×*g* for 10 min at 4 °C. Following centrifugation, the supernatant was passed through a 0.22-μm sterile filter (Steritop™ Millipore, MA, USA). Then, the filtered supernatant was transferred to the upper compartment of an Amicon Ultra-15 Centrifuge Filter Unit (Millipore) and centrifuged at 4000×*g* until the volume of the upper compartment was reduced to ~ 200 μL. The liquid was loaded onto the top of a 30% sucrose/D2O cushion in a sterile Ultra-Clear™ tube (Beckman Coulter, CA, USA) and underwent a 10,000×*g* centrifugation step for 60 min at 4 °C in an Optima L-100 XP Ultracentrifuge (Beckman Coulter) in order to purify the exosomes. Partially purified neuron-derived exosomes were recovered using an 18-G needle diluted in PBS and centrifuged at 4000×*g* at 4 °C in a filter unit until the final volume reached 200 μL. Exosomes were used for downstream experiments or stored at − 80 °C

A Nanosight LM10 System (Nanosight Ltd., Navato, CA) was used to analyze the distribution of vesicle diameters from the exosomes. A transmission electron microscope (TEM; Tecnai 12; Philips, Best, The Netherlands) was used to observe the morphology of the acquired exosomes. Western blotting was used to determine specific exosome surface markers, such as CD9, CD63, and CD81.

### Exosome uptake by microglia

Fluorescent exosome labeling was performed according to the manufacturer’s instructions. A 4-mg/mL Dil solution (Molecular Probes, OR, USA) was added to PBS containing exosomes and incubated. Excessive dye from labeled exosomes was removed by ultracentrifugation at 100,000×*g* for 1 h at 4 °C. These Dil-labeled exosomes were co-cultured with microglia for 24 h and the cells were then washed with PBS and fixed in 4% paraformaldehyde. The uptake of Dil-labeled exosomes by microglia was observed using laser confocal microscopy.

### Vector constructs, lentivirus production, and cell transfections

LV2-mmu-miR-124-3p-mimic vector (miR-124-3p^OE^) and LV2-mmu-miR-124-3p-inhibitor vector (miR-124-3p^KD^) were constructed using lentiviral vectors (GenePharma, Shanghai, China). A negative control was constructed using the LV2 empty lentivirus (miR-NC^OE^ and miR-NC^KD^). Neurons were infected using lentiviral vectors at an appropriate multiplicity of infection. Vectors for the overexpression and shRNA targeting mouse MYH9 using lentiviral gene transfer were constructed by GenePharma (Shanghai, China). The scrambled lentiviral construct was used as a negative control. Microglia were transfected with the lentiviral vectors (shNC, shMYH9, vector, and MYH9).

### In vitro detection of miR-124-3p transfer

Neurons were transfected with 5′-carboxyfluorescein (FAM)-labeled miR-124-3p mimics, miR-124-3p inhibitor, and their corresponding negative controls (GenePharma) with Lipofectamine 3000. After that, exosomes were extracted from the culture medium, divided into four different groups, and added into target microglial cells. After co-incubation, microglial cells were fixed with 4% PFA, permeabilized with 0.05% Trition X-100, and stained with DAPI (Thermo Fisher Scientific). Images were acquired using a confocal microscope to observe green signaling intensity in the target microglial cells.

### Western blot analysis

Proteins were extracted from cells and treated with RIPA lysis and extraction buffers (KeyGen Biotechnology, Nanjing, China). Protein concentration was determined using the BCA method. Equal amounts of protein were separated by SDS-PAGE, transferred to PVDF membranes (EMD Millipore Corp, Burlington, MA), and incubated overnight at 4 °C with primary antibodies followed by blocking with bovine serum albumin (BSA, 5%, v/v). Membranes were then incubated for 2 h at room temperature with the secondary antibody. Immunolabeled bands were visualized using the ECL reagent (Thermo Fisher Scientific) and expression of target protein bands was semi-quantified using ImageJ (National Institutes of Health, Bethesda, MD, USA).

### Quantitative real-time PCR

Total RNA from cells and exosomes was extracted using TRIzol^®^ reagent (Invitrogen, Carlsbad, CA, USA). A reverse transcription system (Toyobo, Osaka, Japan) was used to synthesize complementary DNA (cDNA) and an ABI 7900 fast real-time PCR system (Applied Biosystems, Carlsbad, USA) was used to carry out qRT-PCR with SYBR Green PCR master mix (Applied Biosystems, Foster City, CA). Expression levels were normalized to the internal controls (β-actin or U6) and the 2^−ΔΔCT^ method was used to evaluate the level of relative expression. The specific primers for miR-124-3p, miR-149-3p, miR-218-5p, miR-29c-3p, miR-31, MYH9, U6, and β-actin were purchased from RiboBio Co, Ltd. (Guangzhou, China). The primer sequences are listed in Additional file 1: Table S1.

### miRNA microarray assay

The microRNA arrays for microglia treated with Exos and PBS were performed by OE Biotech Company (Shanghai, China). Each group processed three samples. The fragmentation mixtures were hybridized to an Agilent- Mouse microRNA array 21.0 (8 * 60 K, Design ID:070155). The Affymetrix (Santa Clara, CA, USA) miRNA 4.0 platform was employed for microarray analysis. The sample labeling, microarray hybridization and washing were carried out by the manufacturer’s instructions (Agilent Technologies Inc., Santa Clara, California, USA). Using a fold change cut off value of ≥ 1.5 set for both up- and down-regulated genes to identify different miRNAs expression.

### Luciferase reporter assay

Sequences corresponding to the 3′-UTR of MYH9 mRNA and containing wild-type (WT) or mutated (MUT) miR-124-3p binding sequences were synthesized by GeneScript (Nanjing, China). These sequences were cloned into the FseI and XbaI restriction sites of the pGL3 luciferase control reporter vector (Promega, Madison, WI, USA) to generate the MYH9 3′-UTR reporter constructs (pGL3-WT-MYH9 and pGL3-MUT-MYH9). Microglia were seeded in 24-well plates and incubated for 24 h before transfection. Microglia transfected with miR-124-3p^OE^ or negative control were seeded into 96-well plates and co-transfected with 100 ng of pGL3-WT-MYH9 or pGL3-MUT-MYH9 3′-UTR. Firefly and Renilla luciferase signals were determined using a Dual-Luciferase^®^ Assay Kit (Promega).

### Isolation of RISC-associated RNA

Microglia overexpressing miR-124-3p or miR-NC^OE^ were fixed with 1% formaldehyde followed by chromatin fragmentation, lysed in the NETN buffer, and incubated with Dynabeads Protein A (Invitrogen) supplemented with clone 2A8 antibody (Millipore), anti-Pan-Ago, or IgG control for immunoprecipitation. Proteinase K digestion and phenol/chloroform/isopropyl alcohol were used to release and extract the immunoprecipitated RNA. RNA was isolated by glycogen ethanol precipitation and then treated with DNase I.

### ELISA

To evaluate the expression levels of C1q, TNF-α, IL-1α, IL-1β, and IL-6 in the injured spinal cord, the tissues were isolated there days after SCI. The injured spinal cord was processed using a homogenizer with liquid nitrogen. Lysis buffer, which included 1% Triton X-100, 1-mM EDTA, 1-mM phenylmethylsulphonyl fluoride, 10-mM Tris pH 8.0, 5-μL/mL protease inhibitor, and 150-mM NaCl, was then added into the lysates and incubated for 1 h at 4 °C. The lysates were centrifuged at 3000 rpm for 30 min. According to the manufacturers’ protocols, the collected supernatants were analyzed using ELISA kits to measure the cytokine concentration.

In microglial culture medium, the pro-inflammatory cytokines were measured using ELISA kits according to the manufacturers’ protocols. Optical density or fluorescence was measured with a plate reader.

### Mouse SCI model and experimental groups

The animal protocols were approved by the Animal Committee of the First Affiliated Hospital of Nanjing Medical University. A male mouse (C57BL/6, 6–8 weeks old) SCI model was established. Animals were deeply anesthetized with Ketamine (100 mg/kg) + Xylazine (10 mg/kg) in saline by intraperitoneal (IP) injection. Laminectomy was conducted to expose the spinal cord at T10 and a rod weighing 5 g was dropped from a height of 6.5 cm onto the spinal cord to induce injury using an impactor (RWD, CA, USA, 68097). The muscles were sutured immediately after injury and the wound was closed. Bladders of mice were voided three times a day until the reflexive control of bladder function was restored.

Mice were randomly divided into several groups [n = 8/group for each time point (day 3 and day 28)]. They were subjected to SCI, followed by tail vein injection of exosomes, miR-NC^OE^-Exos, miR-124-3p^OE^-Exos, miR-NC^KD^-Exos, miR-124-3p^KD^-Exos (200 μg of exosome protein precipitated in 200 μL of PBS), or an equal volume of PBS (200 μL) immediately following SCI.

### Functional locomotor scores

The Basso Mouse Scale (BMS) was used to quantify neurological function 1, 3, 7, 14, and 28 days post-injury for locomotion. Scoring ranged from 0 (complete paraplegia) to 9 (normal function). Footprint analysis was performed 28 days post-surgery. Mouse forelimbs (blue) and hind limbs (red) were stained with dyes of different colors. The stride lengths were measured when the mice ran at a constant velocity. The mouse was assessed by two independent examiners blinded to the treatment regimen.

### Swimming test

Swimming test was used to evaluate motor function recovery in the mice post SCI. Mice were trained to swim from one end of the glass tank to the other. Louisville Swim Scale was used to assess the hind limb movement and alternation, forelimb dependency, body angle, and trunk instability. Each mouse was tested twice, and the average score of the two trials is the final score.

### Electrophysiology

According to previous studies [34, 35], electrophysiology was used to analyze motor-evoked potentials (MEPs) in mice. Mice were anesthetized with 10% chloral hydrate solution. Next, applying the stimulation electrode to the rostral ends of the exposed spinal cord, the recording electrode was placed on the biceps femoris flexor cruris, the reference electrode was inserted into the distal tendon of the hind limb muscle, and the ground electrode was placed subcutaneously. A single square wave stimulus (0.5 mA, 0.5 ms, 1 Hz) was applied. The nerve conduction function of the hind limb was detected by peak-to-peak amplitude.

### Nissl staining of spinal cord sections

The cytosolic Nissl substance in spinal cord sections was stained with cresyl violet at Day 28 post-injury. Using the distilled water to wash sections. Then, sections were stained for 10 min in the cresyl violet solution. After rising with distilled water, 95% ethanol was used to differentiate sections washed with xylene and neutral balsam were used to wash and fix. Serious tissue loss of staining was used to identify regions of traumatic injury.

### Immunofluorescence staining

Mouse hearts were perfused with 0.9% saline and 4% paraformaldehyde. Lesion spinal segments were fixed overnight in 4% paraformaldehyde and dehydrated in 15% and 30% sucrose solutions. The spinal segments were frozen and cut into 10-mm-thick sections for experiments. In order to perform tissue immunofluorescence staining, sections were blocked with 10% BSA, incubated overnight at 4 °C with the primary antibodies anti- IBA1, anti-INOS, anti-GFAP, anti-C3, and anti-NF200, and incubated for 1 h with secondary antibodies at room temperature. In order to perform cell immunofluorescence staining, cells were fixed in 4% paraformaldehyde for 30 min, permeabilized with 0.05% Triton X-100, and then blocked with 5% BSA. The cells were then incubated with primary antibodies (anti-IBA1, anti-INOS, anti-GFAP, and anti-C3) overnight at 4 °C, followed by incubation with secondary antibodies. After washing three times with PBS, nuclei were counterstained with DAPI (Thermo Fisher Scientific). Representative immunostaining image of IBA1/INOS on day 3 after injury, and GFAP/C3 in the injured spinal cord lesion areas on day 28 after injury. Fluorescent images were acquired using a fluorescence microscope (AxioVertA1 and ImagerA2).

### Statistical analyses

Experiments were performed in at least three independent biological replicates. Data were expressed as mean ± standard deviation. Statistical analysis was performed using GraphPad software 8.0 and SPSS 25.0. A Student’s t-test for two-group comparisons and one-way or two-way ANOVA for more than two-group comparisons were used to calculate the P-values. A value of *P *< 0.05 was regarded as statistically significant.

## Results

### Identification of primary neuronal cells cultured in vitro

**Fig. 1:**
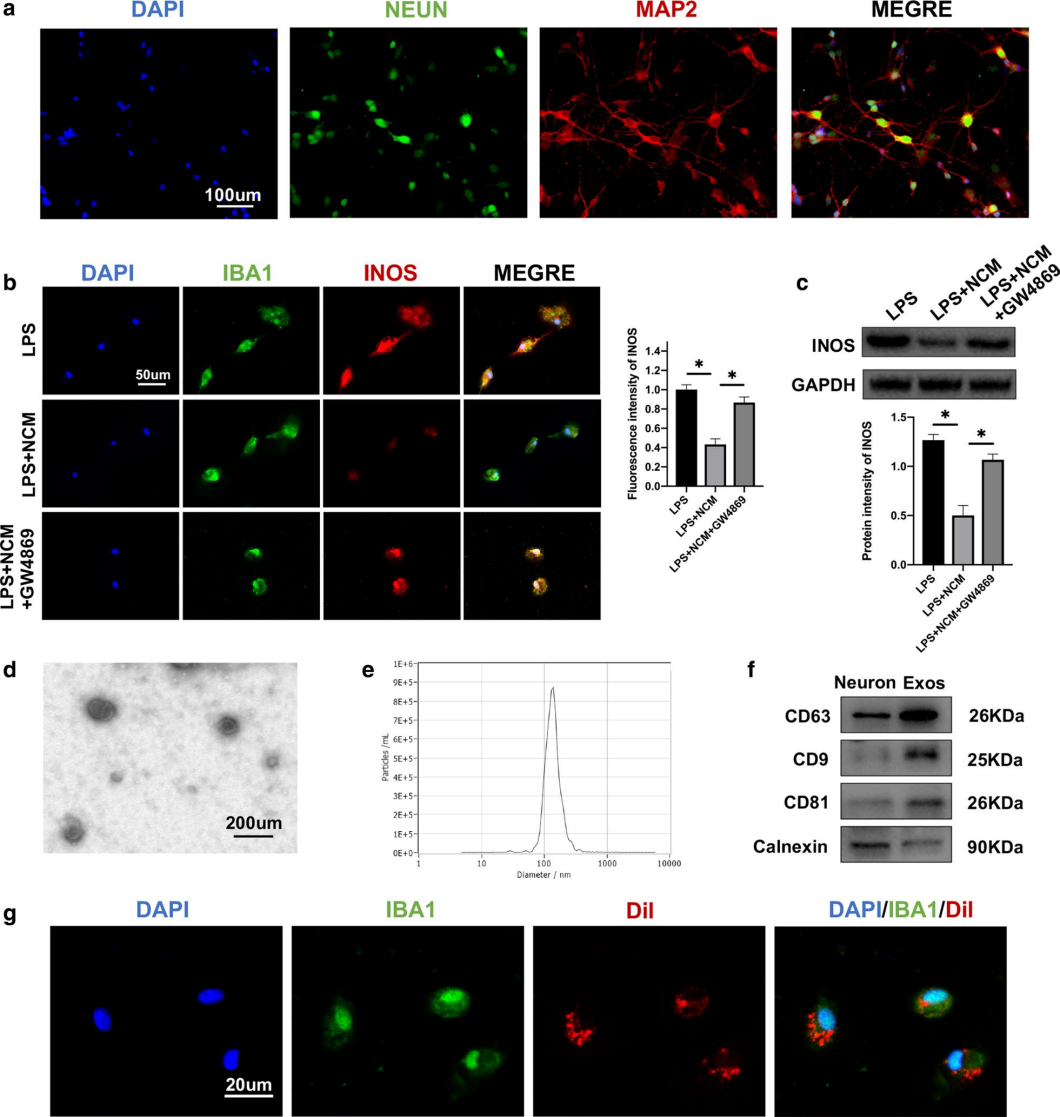
Neuron-derived exosomes suppressed the activation of M1 microglia. **a** Neuronal dendrites and axons were identified by MAP2 and somata by NEUN immunostaining. **b** Representative immunostaining image of IBA1 and INOS in the microglia. **c** The protein expression levels of INOS were detected by western blot in microglia in the different groups. **d** Morphology of exosomes under TEM (50–150 nm). **e** NTA analysis exhibit exosomes size. **f** Western blot analysis of exosome surface markers. **g** The red fluorescence dye Dil-labeled exosomes was uptaken into microglia. *P < 0.05

The primary neurons were demonstrated by immunofluorescence staining of MAP2, a marker of mature neurons. Cells also showed typical neuronal morphology with MAP2-positive axons and dendrites and NEUN-stained somata (Fig. 1a).

### Neuron-derived exosomes suppressed activation of M1 microglia

Initially, neurons were cultured and NCM was collected. Isolated microglia were treated with LPS for 24 h and then co-cultured with NCM. To evaluate microglial activation, M1 microglia were identified by immunofluorescence staining of ionizing calcium binding adaptor molecule 1 (IBA1) and inducible NO (INOS). A significant reduction in expression levels of INOS in the co-cultured group with NCM was compared to the only LPS-microglial medium group. As exosomes play a crucial role in intercellular communication through transfer of genetic material. We supposed it may be related to exosomes secreted by neurons. Therefore, neurons were pre-treated with GW4869 to inhibit exosome secretion. Co-cultured NCM pre-treated GW4869 restored the expression levels of INOS (Fig. 1b). Moreover, INOS western blot analysis also confirmed the immunofluorescence analysis results (Fig. 1c).

### Characterization of neuron-derived exosomes

Exosomes were isolated from the neuron culture supernatant by a combination of centrifugation, ultrafiltration, and ultracentrifugation and analyzed using transmission electron microscopy (TEM), nanoparticle tracking analysis (NTA) and western blot. TEM revealed typical nanoparticles ranging in size from 50 to 150 nm in diameter and NTA exhibited a similar size distribution (Fig. 1d, e). Western blot revealed the presence of exosome surface markers, including CD9, CD63, and CD81, with an absence of calnexin (Fig. 1f). The uptake of Dil-labeled exosomes by microglia was observed (Fig. 1g). These analyses confirmed isolation of exosomes from neuron cultures.

### Transplantation with exosomes promoted functional recovery and reduced lesion volume following traumatic SCI

**Fig. 2:**
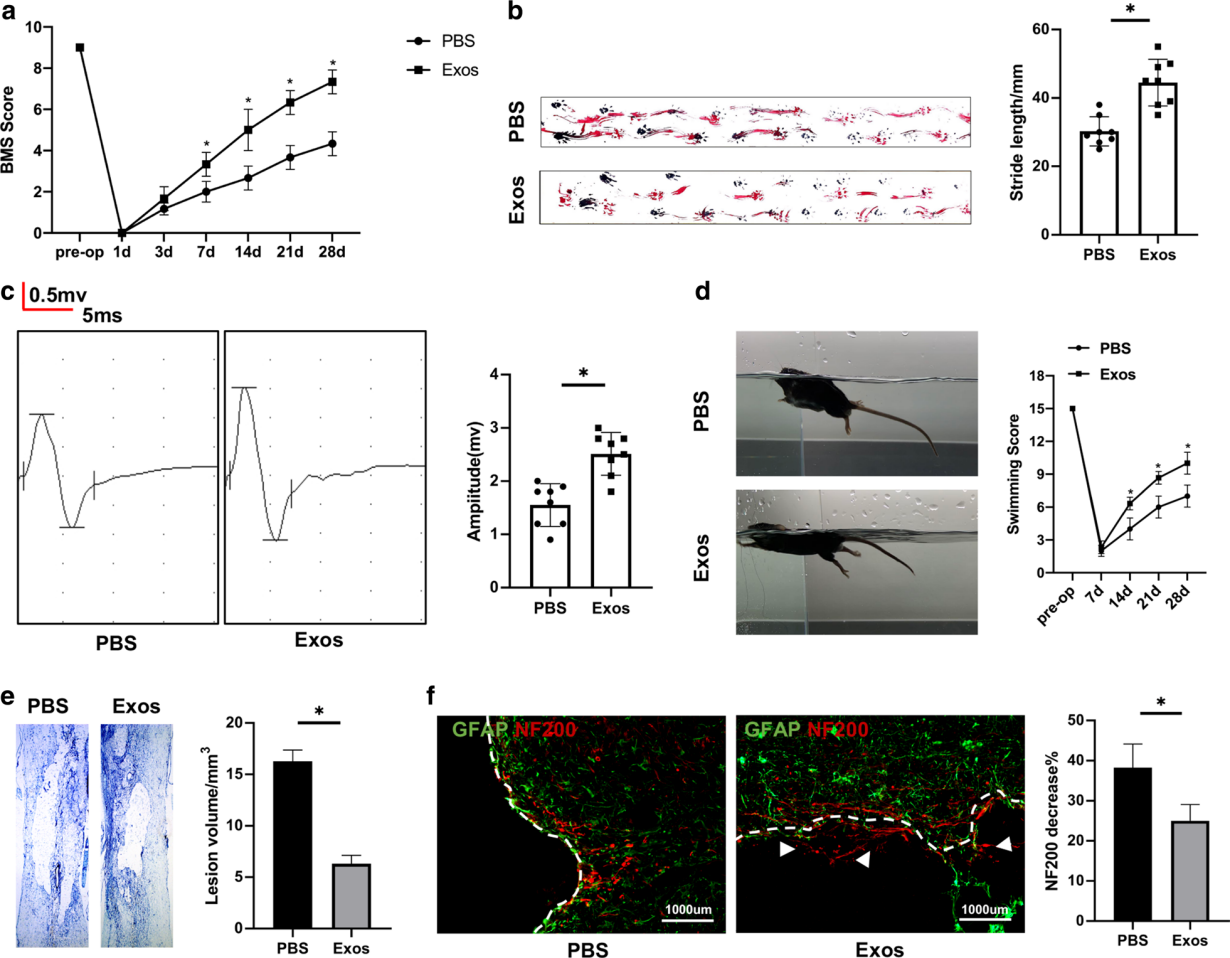
Neuron-derived exosomes promoted functional behavioral recovery after SCI. **a** BMS was used to functionally grade in the PBS and Exos groups at 28 days after SCI for mice. **b** The footprints quantification of mice walking 28 days after SCI. Blue: frontpaw print; red: hindpaw print. **c** MEP was carried out as an electrophysiological assessment in two groups at 28 days after SCI. **d** Photographs of the various degrees of trunk instability (TI) that were observed after SCI surgery, and statistical analysis of the Louisville Swim Scale in the Exos and PBS groups over a 28-day period. **e** Representative Nissl-stained sagittal section of spinal cord. **f** Immunostaining of NF200 and GFAP in the injured areas of spinal cord at 28 days after SCI. *P < 0.05

Initially, BMS scores, gait analysis, electrophysiology analysis and swimming test were used to evaluate motor function after treated with PBS or exosomes. Traumatic SCI was observed on postoperative day 1 in both groups. However, the Exos group exhibited significant improvement in the BMS score compared to the PBS group after SCI (Fig. 2a). Also, gait recovery and improved motor coordination were significantly faster in the Exos group than in the PBS group (Fig. 2b). Moreover, MEP amplitudes were higher in the Exos group was evident when compared to the PBS groups in electrophysiology analysis (Fig. 2c). The Louisville Swim Scale also showed that the mice treated with exosomes exhibited less forelimb dependence, faster hind limb alternation, and a smaller angle between the body and water surface beginning on day 7 post injury (Fig. 2d). Next, Nissl staining revealed a significant loss of tissue in the PBS group than that in the Exos group (Fig. 2e). To further investigate the anatomical basis of the observed locomotor recovery, the density and status of axons in the injured spinal cord were examined. Neurofilaments are qualified as potential surrogate markers of damage to the axon [36]. A 200-kDa neurofilament subunit (NF200) was measured using immunofluorescence to evaluate axonal damage. The decrease in staining against NF200 in the lesion areas compared to the distant area was assessed by average pixel intensity values and was much lower in the Exos group than in the PBS group on postoperative day 28 (Fig. 2f).

### Neuron-derived exosomes suppressed activation of M1 microglia

**Fig. 3:**
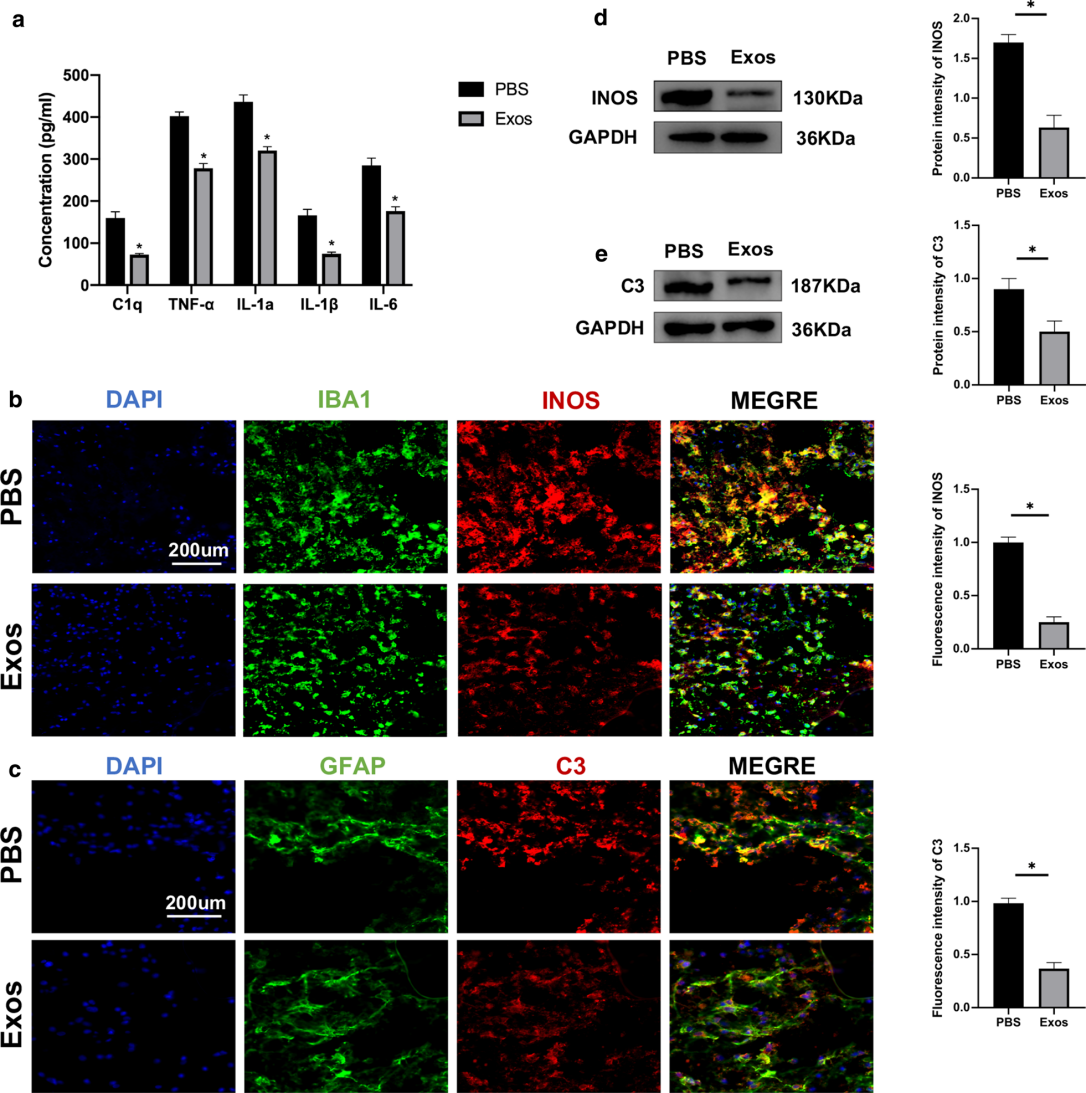
Administration of exosomes following SCI suppressed the activation of M1 microglia and A1 astrocytes in vivo. **a** The concentration of pro-inflammatory cytokines in PBS and Exos groups. **b** Representative immunostaining image of IBA1/INOS in the injured spinal cord lesion areas and the analysis of INOS-positive microglia in the traumatic lesion area. **c** Representative immunostaining image of GFAP/C3 in the injured spinal cord lesion areas and the analysis of C3-positive astrocyte in the traumatic lesion area. **d** Western blot analysis of M1-related proteins in PBS and Exos groups. **e** Western blot analysis of A1-related proteins in PBS and Exos groups. *P < 0.05

Three days after the SCI, ELISA was used to measure the concentration of pro-inflammatory cytokines, including C1q, TNF-α, IL-1α, IL-1β, and IL-6 in the spinal cord of the two groups. The administration of exosomes significantly reduced the concentrations of pro-inflammatory cytokines compared to the PBS group (Fig. 3a). In most pathological conditions M1 microglia are pro-inflammatory [7]. To evaluate the effect of exosomes on M1 microglia, microglia near the injury site were identified using immunofluorescence staining of IBA1, while M1 microglia were identified using immunofluorescence staining of INOS. A significant reduction in the fluorescence intensity of INOS in the traumatic lesion area was found in the exosome treatment group compared to the PBS group (Fig. 3b). Moreover, western blot analysis further confirmed these results (Fig. 3d).

### Neuron-derived exosomes suppressed activation of A1 astrocytes

Pro-inflammatory cytokines that are secreted by activated microglia induce activation of A1 astrocytes [14]. The present work demonstrated that administration of exosomes suppressed the activation of M1 microglia. Therefore, expression of A1 astrocytes was tested to find out if exosomes can suppress activation of A1 astrocytes. A1 astrocytes were identified near the injury site by immunofluorescence staining for complement component 3 (C3) and glial fibrillary acidic protein (GFAP). The fluorescence intensity of C3 was significantly lower in the exosome treatment group compared to the PBS group (Fig. 3c). Moreover, western blot analysis further confirmed these results (Fig. 3e).

### MiR-124-3p is upregulated in exosomes and transferred to microglia by exosomes

**Fig. 4:**
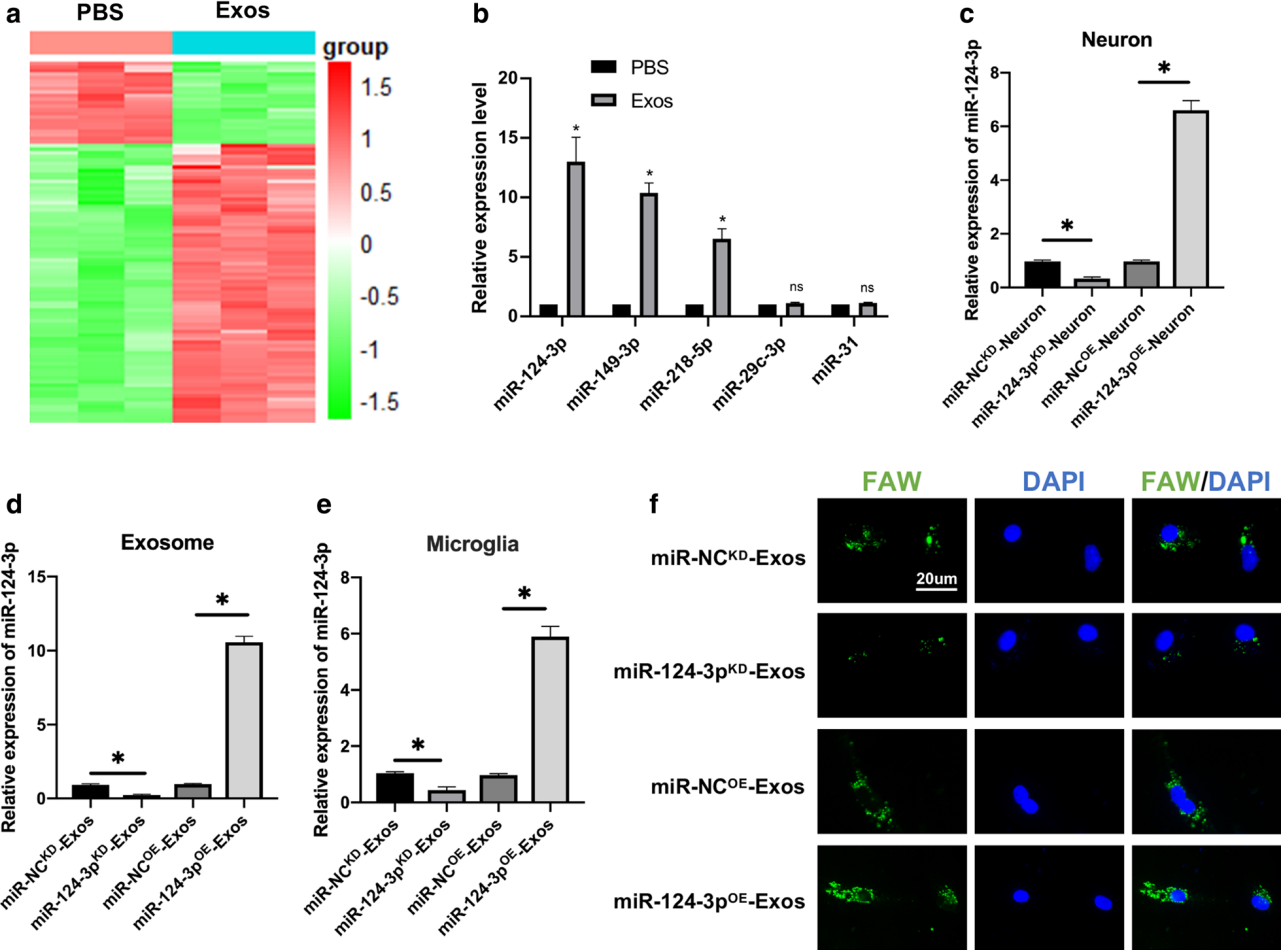
MiR-124-3p is upregulated in Exos and transferred to microglia by exosomes. **a** The 156 upregulated and 35 downregulated miRNAs in Heat map with a ≥ 1.5-fold difference between microglia treated with exosomes and PBS. **b** qRT-PCR was used to compare the top five elevated miRNAs including miR-124-3p, miR-149-3p, miR-218-5p, miR-29c-3p, and miR-31 between microglia treated with exosomes and PBS. **c** miR-124-3p knockdown and overexpression in neurons and the efficiency was confirmed using qRT-PCR. **d** The relative expression level of miR-124-3p in exosomes derived from neurons transfected with miR-124-3p^OE^, miR-NC^OE^, miR-124-3p^KD^ and miR-NC^KD^. **e** Expression level of miR-124-3p in microglia after administering miR-NC^KD^-Exos, miR-124-3p^KD^-Exos, miR-NC^OE^-Exos and miR-124-3p^OE^-Exos. **f** Representative images of FAM-labeled exosomal miR-124-3p internalized by microglia after administration of miR-NC^KD^-Exos, miR-124-3p^KD^-Exos, miR-NC^OE^-Exos and miR-124-3p^OE^-Exos. *P < 0.05, ns indicates no significance

The in vivo and in vitro analyses revealed that exosomes promoted functional recovery and suppressed the activation of M1 microglia and A1 astrocytes. Previous studies have shown that exosomal miRNAs can exert regulatory effects on target cells and may play an important role in regulation of biological function [18, 19, 25]. Then, RNA was isolated from microglia treated with exosomes and PBS and microarray profiling of the miRNAs was carried out. A total of 156 upregulated and 35 downregulated miRNAs in the Exos group were compared to the PBS group (≥ 1.5-fold, P < 0.05) in miRNA microarray analysis (Fig. 4a). Next, the top five upregulated miRNAs were selected, which included miR-124-3p, miR-149-3p, miR-218-5p, miR-29c-3p and miR-31, and qRT-PCR was used to further validate the in vitro results. Three miRNAs, including miR-124-3p, miR-149-3p and miR-218-5p from the five selected above were significantly upregulated in Exos group compared to the PBS group (Fig. 4b). Based on the microarray and qRT-PCR in vitro results, further study concentrated on miR-124-3p because it had a significantly increased expression in microglia. It was then determined whether exosomes suppressed inflammation via transfer of miR-124-3p. It is particularly interesting that miR-124-3p, one of the miRs essential for neuronal identity and function, is highly present in neuronal exosomes [37]. We constructed miR-124-3p overexpression (miR-124-3p^OE^) and knockdown (miR-124-3p^KD^) neurons using a lentiviral-based method as well as the corresponding negative control (miR-NC^OE^ and miR-NC^KD^). The miR-124-3p lentivirus transfection efficiency was confirmed using qRT-PCR (Fig. 4c). Next, exosomes were isolated from neurons and named miR-NC^KD^-Exos, miR-124-3p^KD^-Exos, miR-NC^OE^-Exos, and miR-124-3p^OE^-Exos. A significant decrease in the expression of miR-124-3p in miR-124-3p^KD^-Exos when compared to the miR-NC^KD^-Exos, and an increase miR-124-3p expression in miR-124-3p^OE^-Exos when compared to the miR-NC^OE^-Exos (Fig. 4d). Moreover, miR-124-3p expression level in target microglia in the miR-124-3p^OE^-Exos treatment group exhibited an increase in expression when compared to the miR-NC^OE^-Exos treatment group, and miR-124-3p^KD^-Exos treatment group exhibited a dramatic decrease in expression compared with the miR-NC^KD^-Exos (Fig. 4e). In addition, FAM-labelled immunofluorescence was used to observe exosomal miR-124-3p (Fig. 4f). Similar to the real-time PCR results, the immunofluorescence data demonstrated that after treatment with miR-124-3p^KD^-Exos, miR-124-3p immunofluorescence intensity was significantly lower than that of miR-NC^KD^-Exos. The miR-124-3p^OE^-Exos resulted in high immunofluorescence intensity. These data indicated that neuron-derived exosomal miR-124-3p can be transferred to microglia.

### Exosomes suppressed activation of M1 microglia and A1 astrocytes by delivering miR-124-3p in vivo and in vitro

**Fig. 5:**
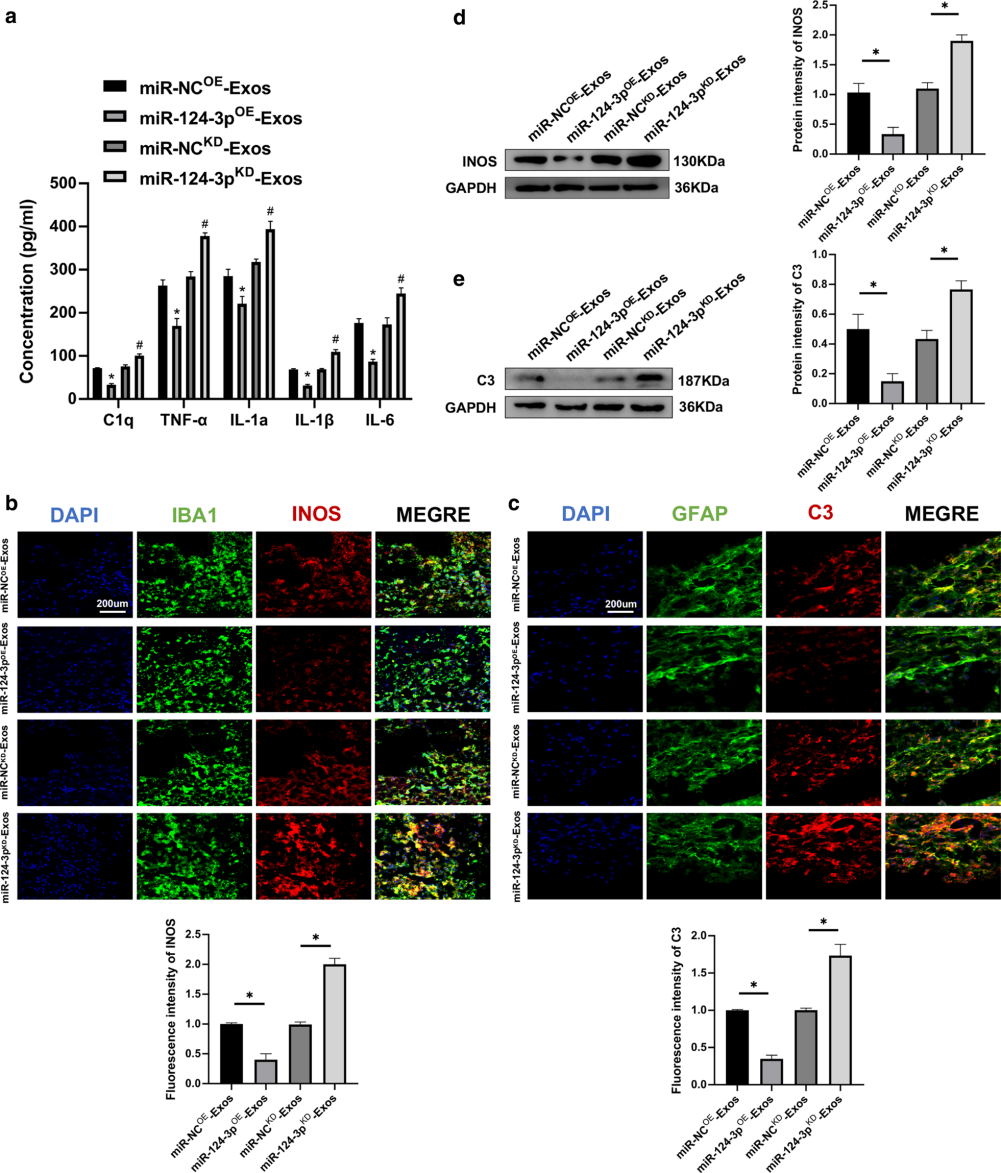
Neuron-derived exosomes suppressed the activation of M1 microglia and A1 astrocytes by delivering miR-124-3p in vivo. **a** The concentrations of pro-inflammatory cytokines in miR-NC^OE^-Exos, miR-124-3p^OE^-Exos, miR-NC^KD^-Exos and miR-124-3p^KD^-Exos groups. **b** Representative immunostaining image of IBA1/INOS in the injured spinal cord lesion areas and the analysis of INOS-positive microglia in the traumatic lesion area. **c** Representative immunostaining image of GFAP/C3 in the injured spinal cord lesion areas and the analysis of C3-positive astrocyte in the traumatic lesion area. **d** Western blot analysis of M1-related proteins. **e** Western blot analysis of A1-related proteins. *P < 0.05 and ^#^P < 0.05

Because the present study was able to demonstrate that neuron-derived exosomal miR-124-3p can be transferred to microglia, we further investigated whether miR-124-3p represented a biological messenger between neurons and microglia and could regulate activation of M1 microglia and induced activation of A1 astrocytes. When compared to miR-NC^OE^-Exos, the concentration of pro-inflammatory cytokines in miR-124-3p^OE^-Exos administration was downregulated in spinal cord tissues. However, the results were the opposite with miR-124-3p^KD^-Exos treatment (Fig. 5a). Using immunostaining, it was found that miR-124-3p^OE^-Exos administration in vivo significantly reduced the fluorescence intensity of INOS and C3 and miR-124-3p^KD^-Exos administration caused an increase in the fluorescence intensity of INOS and C3 (Fig. 5b, c). Western blot analysis confirmed the immunofluorescence results (Fig. 5d, e).

**Fig. 6:**
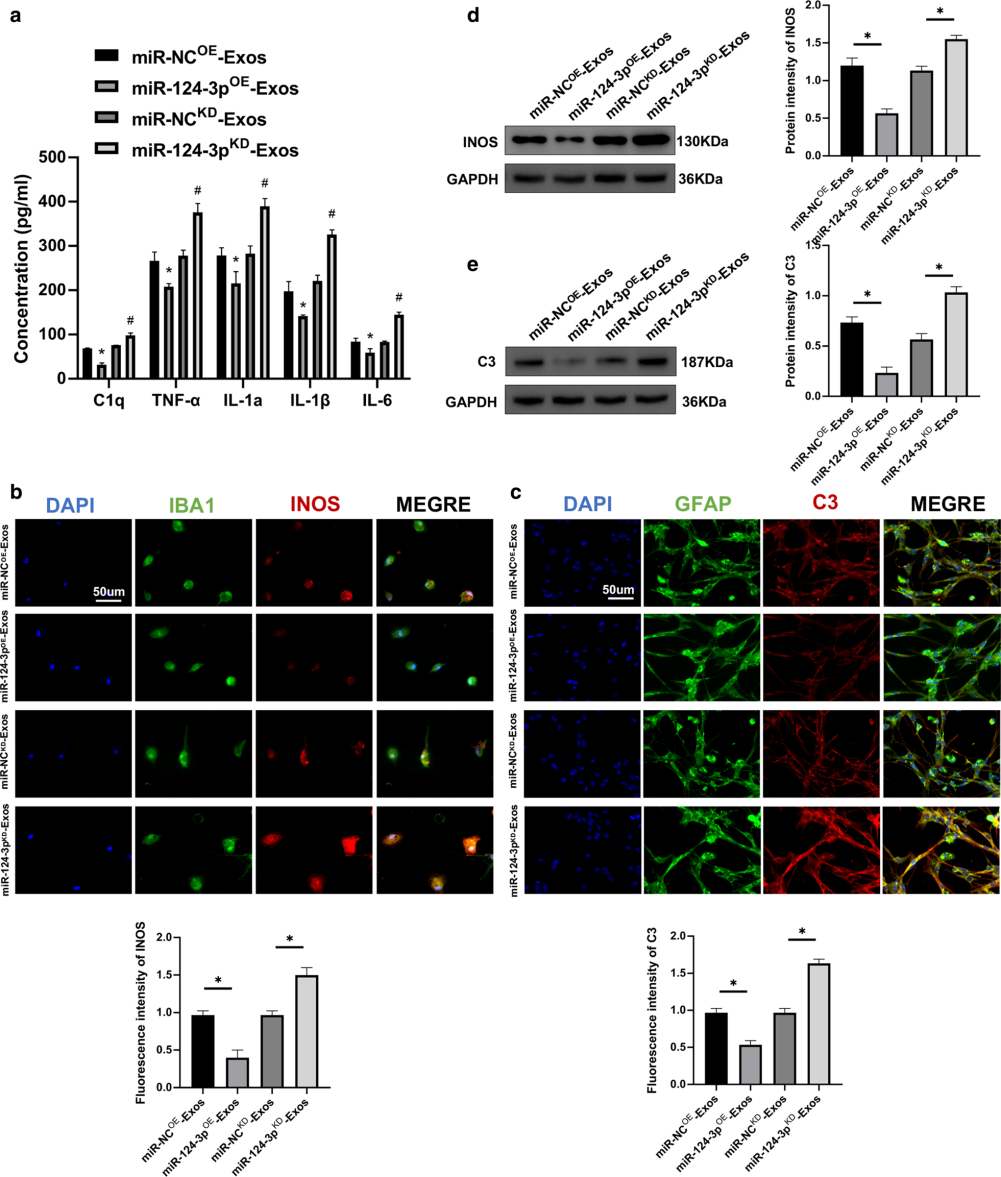
Neuron-derived exosomes suppressed the activation of M1 microglia and A1 astrocytes by delivering miR-124-3p in vitro. **a** The concentrations of pro-inflammatory cytokines in microglia in the miR-NC^OE^-Exos, miR-124-3p^OE^-Exos, miR-NC^KD^-Exos and miR-124-3p^KD^-Exos groups. **b** Representative immunostaining image of IBA1/INOS in the microglia and the analysis of INOS-positive microglia in each group. **c** Representative immunostaining image of GFAP/C3 in the astrocyte and the analysis of C3-positive astrocyte in each group. **d** The protein expression levels of M1-related genes were detected by western blot in microglia in the different groups. **e** The protein expression levels of A1-related genes were detected by western blot in astrocyte in the different groups. *P < 0.05 and ^#^P < 0.05

Furthermore, in vitro experiments in microglia and astrocytes were carried out to explore the mechanism for exosomal shuttling of miR-124-3p. Using ELISAs, it was demonstrated that the pro-inflammatory cytokines were decreased in the miR-124-3p^OE^-Exos group, with the opposite results for administration of miR-124-3p^KD^-Exos (Fig. 6a). Immunofluorescence was applied to determine whether miR-124-3p could exhibit similar effects in vitro. The miR-124-3p^OE^-Exos administration attenuated INOS immunofluorescence in microglia and C3 immunofluorescence in astrocytes compared to miR-NC^OE^-Exos administration. The miR-124-3p^KD^-Exos administration significantly enhanced INOS immunofluorescence in microglia and C3 immunofluorescence in astrocytes when compared to miR-NC^KD^-Exos administration (Fig. 6b, c). Moreover, western blot analysis further confirmed these results (Fig. 6d, e). Taken together, these results demonstrate that exosomes suppressed activation of M1 microglia and A1 astrocytes by delivering miR-124-3p.

### Exosomal miR-124-3p regulates MYH9 by directly targeting 3′-UTR

**Fig. 7:**
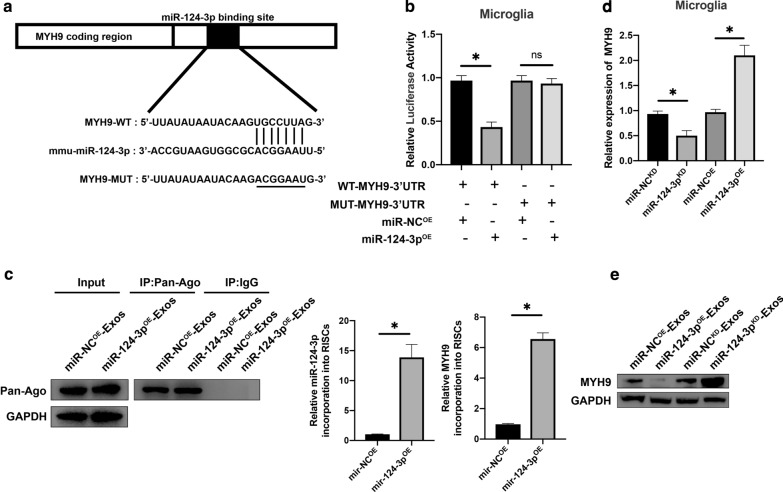
Exosomal miR-124-3p regulates MYH9 by directly targeting the 3′-UTR. **a**, **b** Luciferase reporter assay confirmed that MYH9 was the target gene of miR-124-3p. **c** Immunoprecipitation of the Ago2/RISC (RNA-induced silencing complex) using the Pan-Ago2 antibody in microglia overexpressing miR-NC or miR-124-3p. IgG was used as a negative control and GAPDH was used as an internal control. **d** The mRNA expression level of MYH9 in microglia after miR-124-3p knockdown and overexpression by qRT-PCR. **e** Western blot analysis of MYH9 in microglia after miR-124-3p overexpression and knockdown. *P < 0.05

To further investigate the potential mechanism of action for exosomal miR-124-3p. According to the online database of miRNA targets was used to search the predicted mRNA targets for miR-124-3p. It was found that myosin heavy chain 9 (MYH9) may be the potential target related to inflammation attenuation (Additional file 2: Figure S1). Moreover, MYH9 has been demonstrated to show a positive role in endothelial cell inflammation [38]. To verify that MYH9 3′UTR is a direct target for miR-124-3p, wild-type (WT) and mutated (MUT) 3′-UTR sequences of MYH9 were constructed (Fig. 7a). Transfected microglia were analyzed using a luciferase reporter assay. The relative luciferase activity was decreased when upregulated miR-124-3p was co-transfected with the MYH9 WT luciferase construct, but not with the MUT (Fig. 7b). RNA-ChIP analysis was also used to detect MYH9 mRNA abundance in the Ago2/RNA-induced silencing complex (RISC) after the overexpression of miR-124-3p (Fig. 7c). Enrichment in MYH9 levels that were incorporated into RISC was observed in miR-124-3p overexpressing cells. Then, it was observed that miR-124-3p overexpression decreased MYH9 mRNA and protein expression levels and that knockdown of miR-124-3p increased MYH9 mRNA and protein expression levels (Fig. 7d, e), confirming that MYH9 was the target gene for miR-124-3p.

### MYH9 silencing restore the functional effects of miR-124-3p^KD^-Exos on M1 microglia and A1 astrocytes

**Fig. 8:**
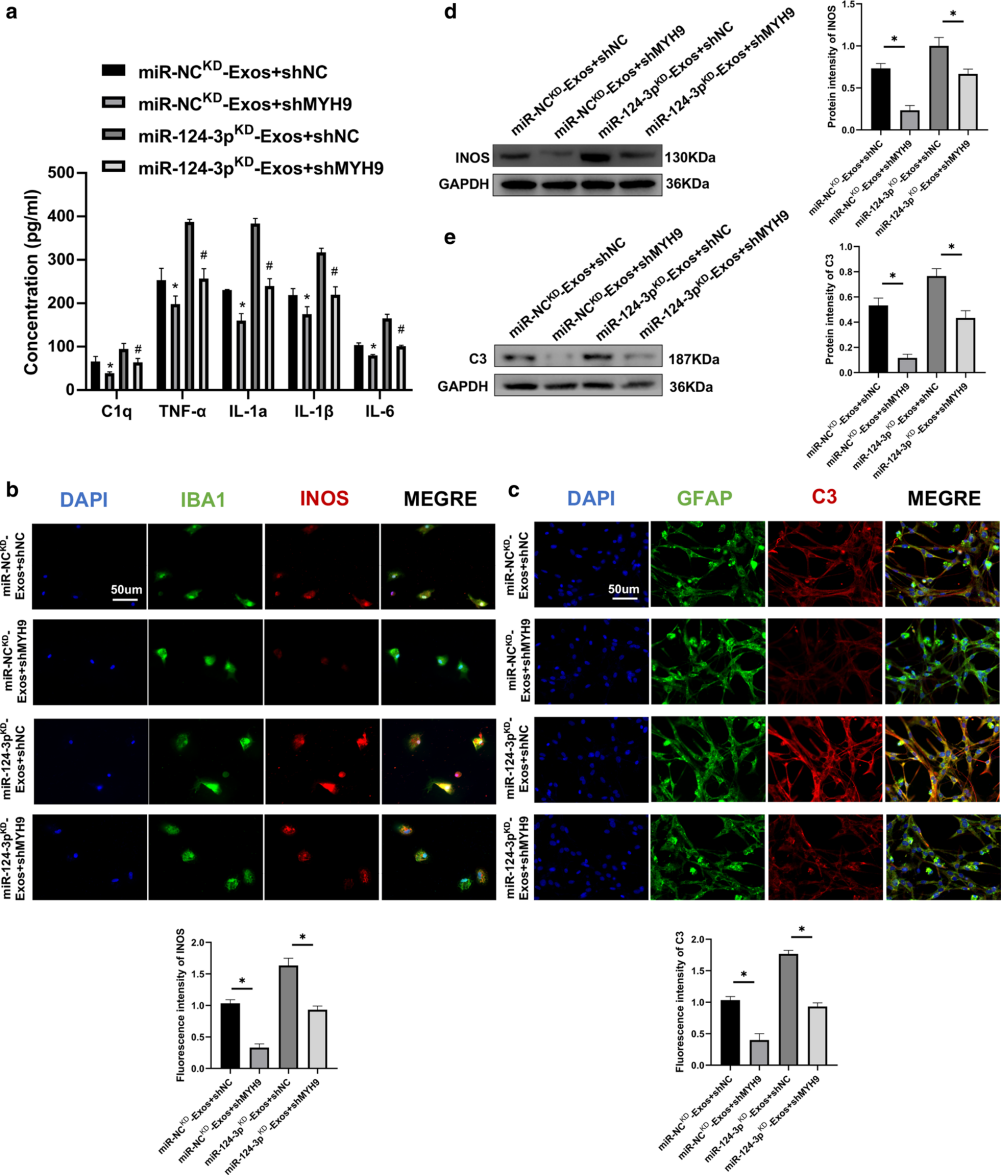
MYH9 silencing restore the functional effects of miR-124-3p^KD^-Exos on M1 microglia and A1 astrocytes. **a** ELISA assays were conducted to evaluate pro-inflammatory cytokines. Rescue experiments for miR-124-3p inhibition were conducted by downregulating MYH9 in microglia. M1 microglia and A1 astrocytes were detected by immunofluorescence (**b**, **c**) and western blot analysis (**d**, **e**). *P < 0.05 and ^#^P < 0.05

To investigate the relationship between exosomal miR-124-3p and MYH9, a series of in vitro rescue experiments were conducted. Endogenous MYH9 expression was silenced in microglia using shRNA technology. Results demonstrated that knockdown of MYH9 inhibited the release of pro-inflammatory cytokines (Fig. 8a). Immunofluorescence results demonstrated that silencing MYH9 suppressed the activation of M1 microglia and A1 astrocytes during co-treatment with miR-124-3p^KD^-Exos (Fig. 8b, c). Furthermore, western blot analysis confirmed these results (Fig. 8d, e). As a result of these rescue experiments, it was demonstrated that shMYH9 in microglia can abolish the inhibitory role of miR-124-3p^KD^-Exos in suppressing the activation of M1 microglia and A1 astrocytes.

### Overexpression of MYH9 abolishes the functional effects of miR-124-3p^OE^-Exos on M1 microglia and A1 astrocytes

**Fig. 9:**
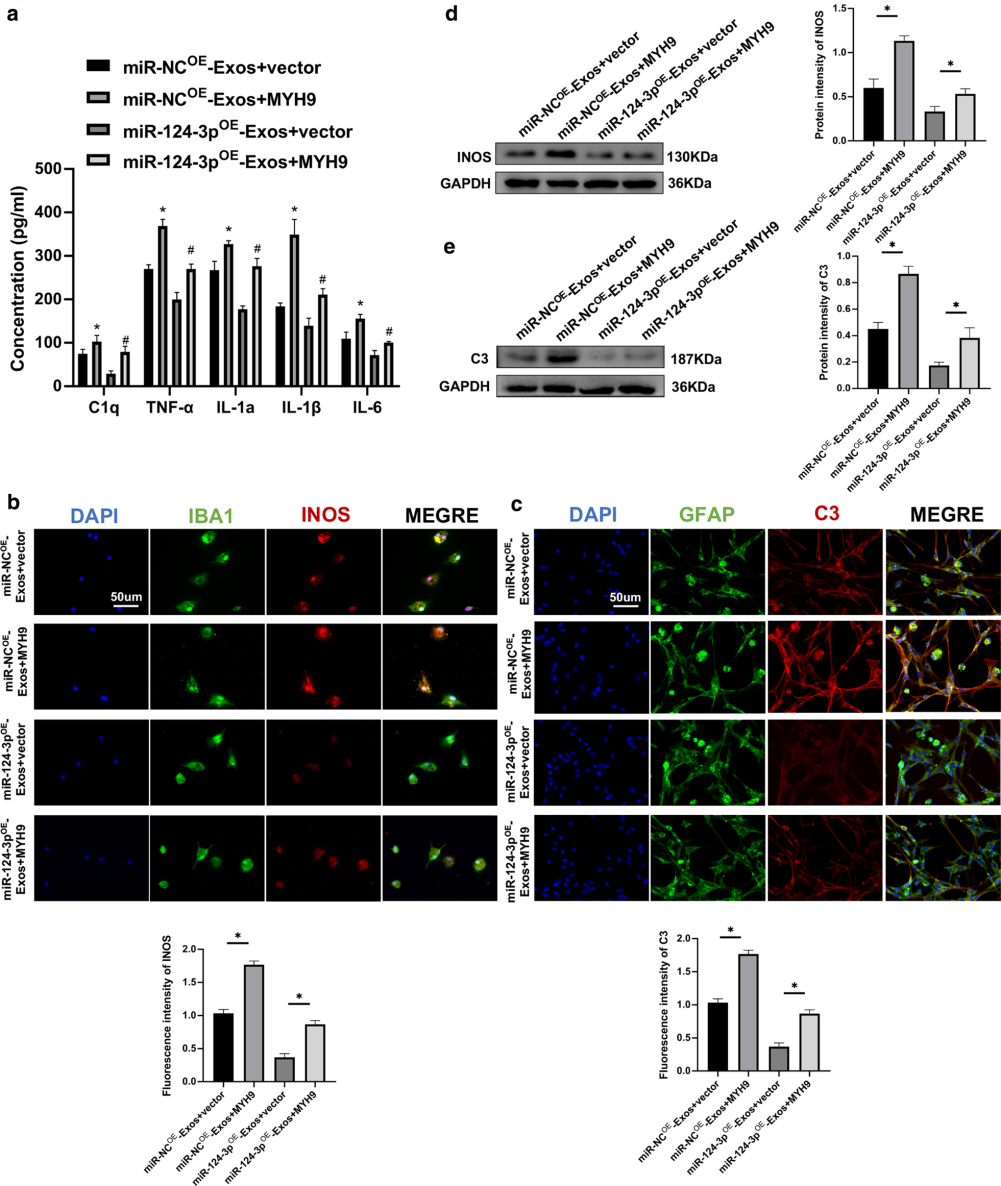
Overexpression of MYH9 abolishes the functional effects of miR-124-3p^OE^-Exos on M1 microglia and A1 astrocytes. **a** ELISA assays were conducted to evaluate pro-inflammatory cytokines. Rescue experiments for miR-124-3p overexpression were carried out by the ectopic expression of MYH9 in microglia. M1 microglia and A1 astrocytes were detected by immunofluorescence (**b**, **c**) and western blot analysis (**d**, **e**). *P < 0.05 and ^#^P < 0.05

MYH9 was overexpressed by transfection with a MYH9 lentivirus in microglia. Results demonstrated that overexpression of MYH9 promoted the release of pro-inflammatory cytokines (Fig. 9a). Immunofluorescence analysis indicated that overexpression of MYH9 reversed the suppression of activation of M1 microglia and A1 astrocytes caused by miR-124-3p^OE^-Exos (Fig. 9b, c). Western blot analysis also confirmed these results (Fig. 9d, e). Therefore, it was concluded that exosomal miR-124-3p suppresses the activation of M1 microglia and A1 astrocytes by targeting MYH9.

### Exosomal miR-124-3p suppresses activation of M1 microglia via the PI3K/Akt/NF-κB pathway

**Fig. 10:**
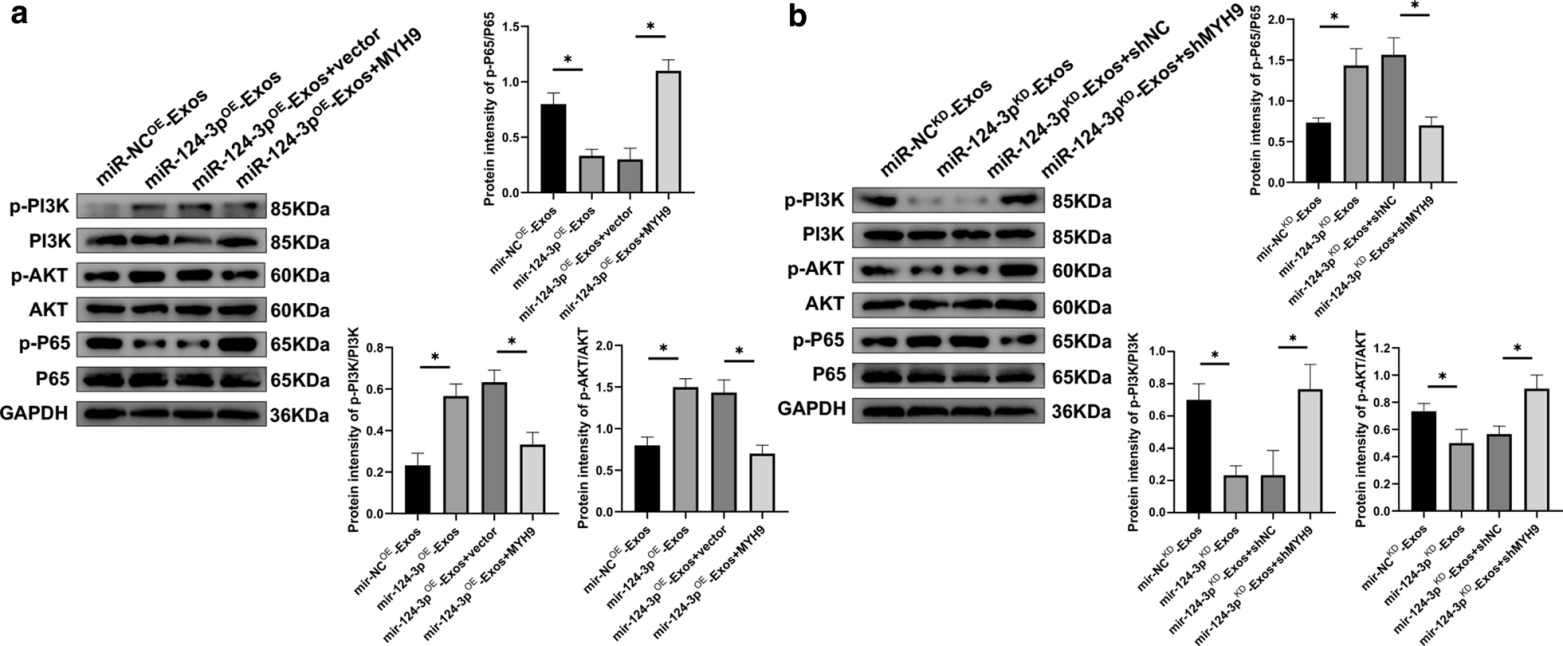
Exosomal miR-124-3p suppressed the activation of M1 microglia through PI3K/AKT/NF-κB signaling cascades. **a** Representative images and quantification of western blots for PI3K/AKT/NF-κB signaling cascades in LPS-stimulated microglia when administrating miR-NC^OE^-Exos, miR-124-3p^OE^-Exos, miR-124-3p^OE^-Exos + vector and miR-124-3p^OE^-Exos + MYH9. **b** Representative images and quantification of western blots for PI3K/AKT/NF-κB signaling cascades in LPS-stimulated microglia when administrating miR-NC^KD^-Exos, miR-124-3p^KD^-Exos, miR-124-3p^KD^-Exos + shNC and miR-124-3p^KD^-Exos + shMYH9. *P < 0.05

MYH9 has been shown to be involved in inflammatory microparticle release in endothelial cells, while MYH9 expression increases TNF-α expression by modulating the PI3K/Akt/NF-κB signaling pathways in endothelial cells [39]. For this reason, it was assumed that exosomes exerted their inhibitory effects on suppressing the activation of M1 microglia via the MYH9/PI3K/Akt/NF-κB signaling pathways. Western blot analysis was performed to compare the expression of major PI3K/Akt/NF-κB pathway members. When treated with miR-124-3p^OE^-Exos, western blot analysis revealed that the expression levels of p-PI3K and p-AKT were markedly upregulated and the expression level of p-P65 was significantly downregulated. However, these effects were all abolished by overexpressing MYH9 (Fig. 10a). The expression levels of p-PI3K, p-AKT, and p-P65 were the opposite with administration of miR-124-3p^KD^-Exos. Similarly, it was reversed when MYH9 was downregulated (Fig. 10b). At the same time, no significant changes were observed in the expression levels of PI3K, AKT, or P65. Taken together, these results indicated that exosomal miR-124-3p suppressed MYH9 by directly targeting its 3′-UTR and thereby modulating the PI3K/Akt/NF-κB pathway.

## Discussion

SCI leads to permanent sensory-motor disabilities with high mortality. Several challenges remain to be overcome to change the incidence, prevalence, and years of life lived with disability [1]. Because exosomes operate on the nanoscale, it has been reported that they can cross the BBB and be absorbed by microglia [40]. Research has applied exosomes as drug delivery vectors through the BBB [31, 41]. M1 microglia is considered pro-inflammatory and increase the secretion of inflammatory cytokines [7, 8]. A1 astrocytes are induced by inflammatory microglia [14]. These states can lead to excessive inflammation and neurotoxic [16, 42].

In our preliminary in vitro experiments, LPS was used to activate microglia in order to mimic the process of neuroinflammation and co-cultured with NCM. Exosomes play an important role in intercellular communication through transfer of genetic material [43]. It has been shown that neurons may exploit the exosomal pathway to maintain homeostasis and regulate cell–cell interactions. The exosomes released are captured by the neighboring cells and exosomal cargoes elicit distinct downstream events [44]. Other studies on exosome secretion from neurons have been conducted with embryonic neurons in culture [24, 45]. It was hypothesized that exosome release is a key mechanism during neurogenesis [46]. We hypothesized that exosomes derived from neurons regulate the activation of microglia. It was then demonstrated that neuron-derived exosomes suppressed the activation of M1 microglia. An SCI model was established in mice. In the preliminary in vivo studies, we demonstrated that transplantation of exosomes promoted functional behavioral recovery and suppressed neuroinflammation in mice following SCI.

As the benefits of exosomes were demonstrated, the study attempted to determine the underlying mechanism that contributes to exosomes effect on promoting functional recovery and suppressing neuroinflammation. Several studies have reported that exosomes derived from CNS exert their biological functions on target cells via delivery of specific miRNAs [24, 44]. Our miRNA microarray analysis showed that miR-124-3p was highly expressed in microglia which treated with exosomes compared to PBS and that exosomal miR-124-3p can be transferred efficiently to the target microglia following treatment with exosomes. A recent study demonstrated that miR-124-3p is highly present in neuronal exosomes and is one of the miRNAs essential for neuronal identity and function [24]. However, a mechanistic study on neuron-derived exosomes, which shuttled miR-124-3p and mediated the effects of suppressing the activation of M1 microglia and A1 astrocytes after SCI, has not been reported. Through a series of in vitro and in vivo experiments, we showed that knockdown of miR-124-3p in exosomes abolished the favorable exosomal effects in the treatment of SCI and that overexpression of miR-124-3p in exosomes demonstrates increased favorable effects. Taken together, we conclude that exosomes enriched with miR-124-3p can suppress the activation of M1 microglia and A1 astrocytes and promote neurological recovery following SCI and that exosomes can act as biological vectors for the delivery of biologically functional miR-124-3p into recipient microglia.

To better understand the underlying mechanism of exosomal miR-124-3p, bioinformatics tools were used to identify the potential target gene for miR-124-3p. As a result, MYH9 was chosen for further study. This target gene was verified using luciferase reporter and RNA-ChIP analyses. Western blot analysis found that the MYH9 protein level was downregulated when overexpressing miR-124-3p and upregulated with the knockdown of miR-124-3p in microglia, which further confirmed that MYH9 was the target downstream gene of miR-124-3p. MYH9 has been demonstrated to show a positive role in cell inflammation [38, 47]. Additionally, MYH9 has been identified to participate in BBB dysfunction in ischemic stroke and to mediate oxidative stress-induced neuronal apoptosis [48, 49]. In our study, to further ensure that MYH9 is the target gene of the identified miRNA, we carried out a series of gain- and loss-of-function experiments. The results demonstrated that knockdown of MYH9 in microglia reversed the unfavorable effects caused by suppressing the expression of miR-124-3p in exosomes, while MYH9 overexpression abolished the beneficial effects observed from overexpression of miR-124-3p in exosomes. Taken together, it can be concluded that exosomal miR-124-3p derived from neurons can suppress the activation of M1 microglia and A1 astrocytes by suppressing microglia-induced neuroinflammation and inhibiting the MYH9 signaling pathway in the process.

Increasing evidence has shown that there is crosstalk between the MYH9 and PI3K/AKT/NF-κB signaling pathways [39]. Suppressing MYH9 signaling can activate the PI3K/AKT signaling pathway and inhibit the NF-κB signaling pathway [50]. Thus, to better understand the signaling pathways after exosome addition, western blot analysis was used to detect the changes in protein levels in the two signaling pathways. The PI3K/AKT pathway was activated and the NF-κB pathway was inhibited following treatment with exosomes and these changes were more evident when administering miR-124-3p^OE^-Exos. However, the effects of activating the PI3K/AKT pathway and inhibiting the NF-κB pathway were partially abolished when administrating miR124-3p^KD^-Exos. These results strongly indicated that exosomal miR-124-3p suppressed the activation of M1 microglia and microglial-induced neuroinflammation by activating the PI3K/AKT and inhibiting the NF-κB signaling cascades, and then suppressing A1 astrocytes.

Although our results implied a crucial role for exosomal miR-124-3p derived from neurons in suppressing microglial-induced neuroinflammation, other possible genes that may act alone or in combination with exosomes to exhibit therapeutic effects cannot be ruled out. The precise mechanism of action of exosomes derived from neurons in the promotion of functional behavioral recovery after SCI in mice will be explored in future studies.

In summary, this study showed that exosomes derived from neurons can suppress the activation of M1 microglia, suppressing microglial-induced neuroinflammation, and then suppressing A1 astrocytes. As these nano-sized exosomes show promising potential as an effective intervention for the delivery of therapeutic administration of miRNAs into the injured spinal cord across BBB, the combination of miRNAs and neuron-derived exosomes may provide a minimally invasive approach for the treatment of SCI.

## Conclusions

Our study highlighted the underlying mechanism by which cell-free exosomes derived from neurons promote functional behavioral recovery by shuttling miR-124-3p following SCI in mice. The enriched levels of exosomal miR-124-3p improved the therapeutic potential by suppressing the activation of M1 microglia, reducing neuroinflammation to suppress A1 astrocytes, and by the MYH9/PI3K/AKT/NF-κB signaling pathway. A combination of miRNAs and neuron-derived exosomes may be a promising, minimally invasive approach for the treatment of SCI.

## Supplementary information

**Additional file 1: Table S1**. The primer sequences used in this work.

**Additional file 2: Figure S1**. Overview of bioinformatics analysis showing MYH9 as a downstream target of miR-124-3p.

## Data Availability

Most of the datasets supporting the conclusions of this article are included within this article and the additional files. The datasets used or analyzed during the current study are available on reasonable request.

## Abbreviations

BBB: Blood–brain barrier
CNS: Central nervous system
Exos: Exosomes derived from neuron
MEP: Motor-evoked potential
miRNA: MicroRNA
SCI: Spinal cord injury
MYH9: Myosin heavy chain 9
LPS: Lipopolysaccharide
NO: Nitric oxide
ROS: Reactive oxygen species
MCM: Microglia-conditioned medium from LPS-treated
NCM: The neuron-conditioned medium
OPCs: Oligodendrocyte precursor cells
C3: Complement component 3
GFAP: Glial fibrillary acidic protein
IBA1: Ionizing calcium binding adaptor molecule 1
INOS: Inducible NO
TEM: Transmission electron microscope
NTA: Nanoparticle tracking analysis
qRT-PCR: Quantitative real-time PCR
RISC: RNA-induced silencing complex
