# Supplementary figures and images for: Neuron-derived exosomes-transmitted miR-124-3p protect traumatically injured spinal cord by suppressing the activation of neurotoxic microglia and astrocytes
Source: J Nanobiotechnology. 2020 Jul 25;18:105. doi: 10.1186/s12951-020-00665-8 (PMC7382861; doi:10.1186/s12951-020-00665-8)

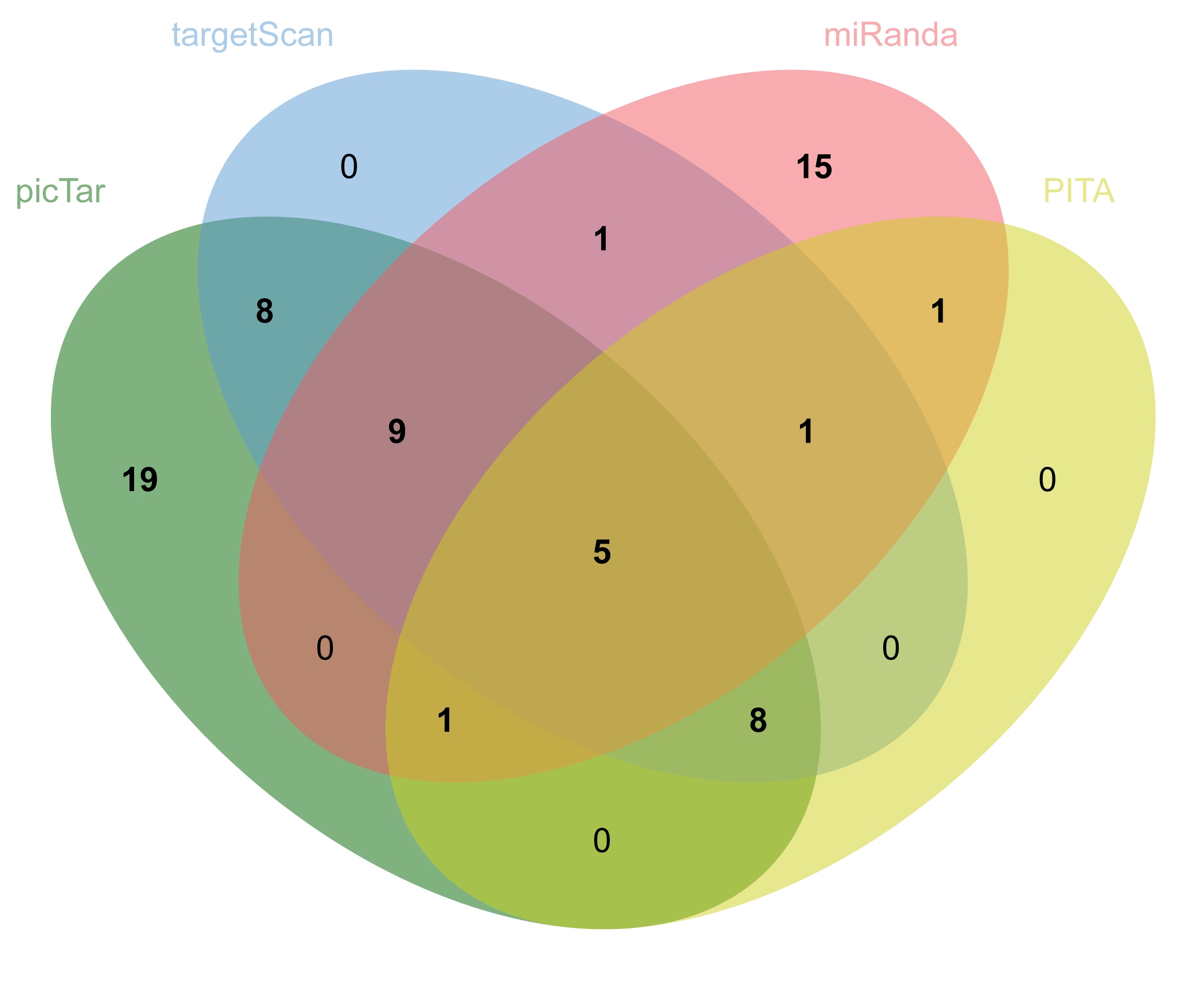

Supplement: Supplementary file 2 — Additional file 2: Figure S1. Overview of bioinformatics analysis showing MYH9 as a downstream target of miR-124-3p. [file 12951_2020_665_MOESM2_ESM.tif]
